# COVID-19 Vaccine: A Survey of Hesitancy in Patients with Celiac Disease

**DOI:** 10.3390/vaccines9050511

**Published:** 2021-05-16

**Authors:** Andrea Costantino, Matilde Topa, Leda Roncoroni, Luisa Doneda, Vincenza Lombardo, Davide Stocco, Andrea Gramegna, Claudio Costantino, Maurizio Vecchi, Luca Elli

**Affiliations:** 1 Gastroenterology and Endoscopy Unit, Foundation IRCCS Ca’ Granda Ospedale Maggiore Policlinico, 20214 Milan, Italy; leda.roncoroni@unimi.it (L.R.); vincenza.lombardo@policlinico.mi.it (V.L.); maurizio.vecchi@unimi.it (M.V.); 2 Department of Pathophysiology and Transplantation, University of Milan, 20124 Milan, Italy; matilde.topa@unimi.it (M.T.); andrea.gramegna@unimi.it (A.G.); 3 Department of Biomedical, Surgical and Dental Sciences, University of Milan, 20124 Milan, Italy; luisa.doneda@unimi.it; 4 Department of Mathematics, Politecnico di Milano, 20124 Milan, Italy; davide.stocco@polimi.it; 5 Internal Medicine Department, Respiratory Unit and Cystic Fibrosis Adult Center, Foundation IRCCS Ca’ Granda Ospedale Maggiore Policlinico, 20124 Milan, Italy; 6 Department of Health Promotion Sciences, Maternal and Infant Care, Internal Medicine and Excellence Specialties “G. D’Alessandro”, University of Palermo, 90145 Palermo, Italy; claudio.costantino01@unipa.it; 7 Center for Prevention and Diagnosis of Celiac Disease, Foundation IRCCS Ca’ Granda Ospedale Maggiore Policlinico, 20124 Milan, Italy

**Keywords:** COVID-19 vaccines, COVID-19, vaccine hesitancy, celiac disease, vaccines

## Abstract

(1) Background: COVID-19 vaccination campaigns offer the best hope of controlling the pandemic. However, the fast production of COVID-19 vaccines has caused concern among the general public regarding their safety and efficacy. In particular, patients with chronic illnesses, such as celiac disease (CD), may be more fearful. Information on vaccine hesitancy plays a pivotal role in the development of an efficient vaccination campaign. In our study, we aimed to evaluate COVID-19 vaccine hesitancy among Italian CD patients. (2) Methods: an anonymous questionnaire was sent to CD patients followed at our tertiary referral center for CD in Milan, Italy. Patients were defined as willing, hesitant and refusing. We evaluated the reasons for hesitancy/refusal and the possible determinants, calculating crude and adjusted odds ratios [AdjORs] with 95% confidence intervals [CIs]. (3) Results: the questionnaire was sent to 346 patients with a response rate of 29.8%. Twenty-six (25.2%) of the 103 respondents were hesitant, with a total refusal rate of 4.8%. The main reason was fear of adverse events related to vaccination (68.2%). Among hesitant patients, 23% declared that their opinion was influenced by their CD. The determinants positively influencing willingness to be vaccinated against COVID-19 were adherence to a GFD, perception of good knowledge about COVID-19 and its vaccines, and a positive attitude to previous vaccines (AdjOR 12.71, 95% CI 1.82–88.58, AdjOR 6.50, 95% CI 1.44–29.22, AdjOR 0.70, 95% CI 0.11–4.34, respectively). (4) Conclusions: CD patients should be vaccinated against COVID-19 and a specific campaign to address the determinants of hesitancy should be developed.

## 1. Introduction

Coronavirus disease 2019 (COVID-2019), caused by the SARS-CoV-2 virus, has resulted in a global healthcare crisis and the deaths of more than three million people worldwide [1]. This has led to many challenges, including restricted social life based on social distancing, the need to stay at home (giving rise to smart working and home-schooling), and disruption of national and household economies. Vaccines offer the most promising solution since they provide individual and population-level immunity, supporting the resumption of normal social and economic activity. To date, the European Medicines Agency (EMA) and the Italian Agency have approved four vaccines four vaccines against COVID-19: BNT162b2 (Pfizer/BioNTech, New York, NY, US), mRNA-1273 (Moderna, Cambridge, MA, US), Ad26.COV2.S/JNJ-78436735 (Johnson & Johnson, New Brunswick, NJ, US) and AZD1222 (AstraZeneca, Cambridge, UK) and AZD1222 (AstraZeneca, Cambridge, UK) [2,3]. The latter has not yet been approved by the U.S. Food and Drug Administration (FDA) [4]. The development of these vaccines within 1 year has been extremely fast. Such unprecedented speed has led to concerns by some about the safety of the vaccines [5,6,7]. Vaccine hesitancy is defined as a delay in acceptance or refusal of vaccine despite availability of vaccine services [8].

Celiac disease (CD) is an autoimmune disorder that affects approximately 1% of the global population, and is caused by an autoimmune reaction evoked by gluten ingestion in genetically susceptible individuals. Adherence to a gluten-free diet (GFD) is an effective treatment in most patients. Compared to the general population, those affected by chronic illnesses such as CD may be more apprehensive about COVID-19, although recent data are reassuring [9,10,11,12,13,14]. A questionnaire was sent to CD patients, asking whether the COVID-19 pandemic made them feel more vulnerable because of their CD, with 56.6% answering negatively; elderly, women, and patients with other comorbidities were the most apprehensive [15]. 

It is uncertain if CD patients have an increased risk of infection because of factors such as defective nutritional status, increased intestinal permeability, and hyposplenism [16], and if they have a lower immunogenic response to vaccination. Some inconclusive findings suggest that CD patients may develop lower immunogenicity after HBV vaccination [16]. Misinformation on the internet and fake news about CD and vaccines can have a dramatic effect even though several scientific societies, including the Society for the Study of Celiac Disease, have clearly supported the administration of COVID-19 vaccines in the CD population [17]. 

Italy began its COVID-19 vaccination campaign at the end of December 2020, when the first vaccines were administered to frontline healthcare workers and nursing home staff and residents. Since 20 February, the campaign was extended to the general public, targeting the priority groups of those over 80 and those working in key sectors including schools, universities, prisons, and the armed forces. At the end of February, 4.9% of the Italian population was vaccinated against COVID-19 with at least one dose, with 2.3% fully vaccinated [18]. Since March, vaccination has been offered to those at very high risk of becoming severely ill with COVID-19, followed by the general population according to age and to comorbidities.

The aim of our study was to evaluate acceptance of COVID-19 vaccination among patients with CD. We investigated the possible determinants of COVID-19 vaccination hesitancy such as CD, attitude to previous vaccinations, lifestyle, health-related behaviors and attitudes, and sociodemographic data.

## 2. Materials and Methods

### 2.1. Study Design

Between 22 February and 26 February 2021, an anonymous questionnaire was sent to a mailing list of CD patients followed at the tertiary level of Celiac Disease Centre of the Foundation IRCCS Ca’ Granda Ospedale Maggiore Policlinico in Milan, Italy. The questionnaire was adapted from a previously validated questionnaire on vaccine hesitancy and sent to patients as a link in an email. It was developed online using the EUSurvey platform by our center. Unlike other such tools, the EUSurvey platform does not allow identification of the user through IT tracking and does not use profiling cookies. Completion of the web-based survey did not result in any benefit or financial compensation for respondents. 

The questionnaire investigated three areas: sociodemographic data, celiac disease-related and lifestyle data, and attitude to vaccinations in general and to the SARS-CoV-2 vaccine in particular. The questionnaire was divided into 10 sections (see Supplementary Materials). A negative answer to the question: “Would you accept vaccination against COVID-19 tomorrow?” was considered hesitancy against COVID-19 vaccination. A negative answer to the question “If you answered no, would you eventually accept it in the future when more data are available?” was considered vaccine refusal. 

Hesitant patients were asked about their reasons and whether CD influenced their decision. CD patients were also asked about their perceptions of COVID-19, specifically if they thought that due to their CD, they were more likely to get the disease and it would be more severe.

The study was approved by our local ethics committee. Patients provided informed consent before completing the questionnaire.

### 2.2. Statistical Analysis

Absolute and relative frequencies were calculated for the categorical (qualitative) variables, and quantitative variables were summarized by their means. All variables found to have a statistically significant association with vaccination hesitancy/refusal in the univariate analysis were included in a multivariate backward stepwise logistic regression model. All variables with *p* ≤ 0.20 were selected in the multivariate model, to guarantee a more conservative approach. The crude odds ratio (crude OR) and the adjusted OR (AdjOR) with 95% confidence intervals (CIs) were also calculated in the logistic regression model. The level of significance chosen for the multivariate logistic regression analysis was 0.05 (two-tailed). The correlation and dependency in the dichotomous variables CAM and vaccination were analyzed using the phi coefficient and test of association. All data were analyzed using the statistical software R (R Core Team, R version R version 4.0.4, Boston, MA, USA).

## 3. Results

The survey was sent to 346 CD patients of whom 103 responded, giving a response rate of 29.8%. The baseline characteristics of the respondents are given in Table 1. 

**Figure 1:**
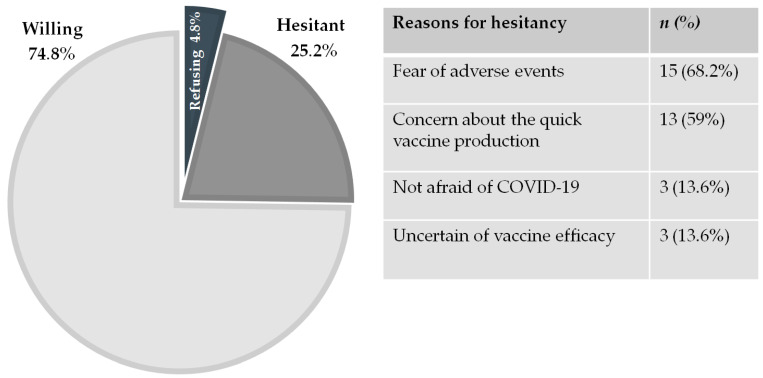
Willingness and hesitancy regarding COVID-19 vaccination in celiac disease patients and reasons for hesitancy.

Twenty-six (25.2%) of the 103 patients were hesitant. Of these patients, five were refusing, for a total refusal rate of 4.8% (5/103) (Figure 1). The main reasons for hesitancy were fear of adverse events and/or distrust of the fast vaccine production (68.2% and 59%, respectively), while 13.6% of hesitant patients were not afraid of COVID-19 and 13.6% thought the vaccine would not be efficient in protecting against disease (Figure 1). Six hesitant patients (23%) said that their decision was influenced by their CD. In contrast, among willing patients, 3% regarded CD as a reason for priority vaccination. Nineteen (18.4%) thought they had a higher risk of experiencing adverse events following COVID-19 vaccination because of their CD. 

Analysis of the determinants of vaccine hesitancy showed that age, expressed as a continuous variable, was not associated with vaccine willingness (*p* = 0.91). Similarly, the difference between men and women was not significant (*p* = 0.13). The AdjOR showed significant positive associations between willingness (shown by 74.8% of CD patients) and a positive attitude to vaccinations in general (AdjOR 16.48, 95% CI (3.34–81.31). Other significant determinants influencing attitude to COVID-19 vaccines were adherence to a GFD (AdjOR 12.71, 95% CI 1.82–88.58) and perception of a good knowledge of COVID-19 and vaccines against COVID-19 (AdjOR 6.50, 95% CI 1.44–29.22) (Table 2). There was negative correlation between a positive attitude to complementary and alternative medicines (CAM) and a positive attitude to general vaccinations, measured using the phi coefficient, a measure of association for two binary variables (phi = −0.399, *p* = 0.00003).

Considering COVID-19 perception among CD patients, 21.4% thought they had a higher risk of contracting COVID-19 due to CD, while 26.2% thought the risk of more severe COVID-19 was greater because of their CD (Table 3).

## 4. Discussion

### 4.1. Impressions

Vaccine hesitancy poses a threat to efforts to control the COVID-19 pandemic through vaccination campaigns. Knowledge of the reasons and determinants significantly affecting willingness to receive a vaccine may guide vaccination campaign strategies. 

In our study, the percentage of patients completely against the new COVID-19 vaccines was interestingly low (refusal rate of 4%), compared with data in a recent global survey of a random population sample in 19 different countries, which showed 8.1% of participants were completely opposed to a COVID-19 vaccine [7]. Hesitancy/refusal was also much lower than that found by a recent COVID-19 vaccine hesitancy survey of an Italian cohort (34.8% vs. 17.6%) [19]. 

The reasons for vaccine hesitancy and refusal were mainly fear of adverse events and concerns about the fast vaccine production processes. It is noteworthy that 27.2% of hesitant patients declared their fears of lack of efficacy and of adverse events were influenced by their CD. A small group of patients (3%) thought CD caused increased risk and was therefore a reason for receiving priority vaccination. 

The negative association found between attitude to vaccination in general and use of CAM has been reported in other studies. CAM users may believe that vaccines and other drugs commonly prescribed by physicians are harmful and instead use alternative medicines and practices such as acupuncture, chiropractic, and herbal medicines. It is presumed that CAM practitioners, such as chiropractors and naturopaths, advise their clients against vaccines [20]. 

The determinants positively influencing willingness to be vaccinated against COVID-19 were adherence to a GFD, perception of good knowledge about COVID-19 and its vaccines, and a positive attitude to previous vaccines. Adherence to GFD positively influenced willingness to be vaccinated against COVID-19. This is not surprising as compliant CD patients are more likely to look after their health and try to prevent diseases, for instance, through vaccination. The perception of having good knowledge about COVID-19 and its vaccines was associated with higher acceptance of vaccination. This could encourage specialists and general practitioners to improve campaigns by providing information on the safety and efficacy data of COVID-19 vaccines, in order to improve people’s awareness. An association between a positive attitude to vaccines in general and COVID-19 vaccine willingness was observed. Patients with CD showed a similar good acceptance of previous vaccines (e.g., HBV, HPV, MMR, DTP, flu). This finding is comparable to the result in a general Italian population who previously completed the same validated questionnaire (82.6%) [21]. That study reported that of 299 enrolled subjects, 12.7% were hesitant about vaccination and 4.7% were refusing.

### 4.2. Vaccines in Celiac Disease Patients

In our study, one out of every four hesitant patients stated his hesitancy was influenced by CD and about one in five patients felt more vulnerable to adverse events following COVID-19 vaccination because of CD.

The high prevalence of CD has led to the study of many aspects of this common autoimmune disorder, included the vaccine immunogenicity in CD patients, focusing on the possible reasons underlying a reduced vaccine response in some cases. It has been demonstrated that after vaccination against HBV, CD patients produce fewer protective antibodies than the general population [22]. Park et al. showed that a significantly greater percentage of children with CD did not respond to HBV vaccination in comparison with age-matched controls (53.9% vs. 11.1%, *p* < 0.05). However, all of the children responded to the other common childhood vaccines (HBV, tetanus, rubella, *Haemophilus influenzae* type b). According to these results, the authors hypothesized that immunogenicity of HBV vaccine may be influenced by HLA haplotypes [23]. The same results have been found by Zingone et al. who evaluated the response to HBV vaccine in relation to gluten consumption in patients with CD and in a control group of healthy subjects. [24]. According to Nemes et al., the importance of disease activity in vaccination failure may play a pivotal role rather than the specific HLA alleles. [25]. A further study showed that adherence to GFD may increase the immune response to HBV vaccination in CD patients [26]. 

Regarding vaccinations and autoimmune disorders, some studies have focused on possible immune modulation leading to an increased risk of developing diseases such as CD or inflammatory bowel disease [27]. In 1995, Thompson et al. performed the first study aiming to retrospectively evaluate the influence of measles vaccination on the development of intestinal autoimmune disorders, but no difference in CD prevalence was found between the two groups of vaccinated individuals and a birth cohort of unvaccinated subjects [28]. To date, no studies have found any correlation between vaccination and autoimmune disease development. Regarding the fear of adverse events after COVID-19 vaccination because of CD, no increased risk of adverse reactions to previous vaccines in CD patients has been described [29]. 

The beginning of COVID-19 vaccination campaigns worldwide has prompted the main medical organizations focused on CD, such as the Society for the Study of Celiac Disease, to comment on the lack of contraindications to the use of available COVID-19 vaccines in CD patients [17]. Likewise, the authors strongly recommended COVID-19 vaccination in every CD patient, to reduce the morbidity and mortality associated with COVID-19, even if it has not been indicated to have an increased risk of severe outcomes in CD patients [9,10,11,12,13,14,30]. However, we do not know if CD patients should be encouraged to get vaccinated against COVID-19 with priority because of their chronic autoimmune disorder. Indeed, a presumed major risk of infections in CD patients has been previously showed by some studies and specifically, connected to hyposplenism [31,32,33]. As regards to *Streptococcus pneumoniae* infection, after the introduction of conjugate vaccines, the rate of pneumococcal disease decreased considerably among children. Nevertheless, several reports of pneumococcal infection and fatal septicemia have been described in a number of celiac patients, particularly in the presence of spleen hypofunction. Therefore, many authors suggested that pneumococcal vaccination should be administered to all CD patients. Authors have also proposed to investigate splenic function in CD patients at high risk of hyposplenism (e.g., concomitant autoimmune disorders, old age at diagnosis, previous history of major infections/sepsis or thromboembolism, and/or spleen atrophy) [31]. Signally, CD appears to be the most frequent pathology associated with functional hyposplenism [16,32,33].

### 4.3. Strenghts and Limitations

Limitations of our study include a possible selection bias as those who filled in the questionnaire may have had a more favorable attitude to vaccines than those who did not. Another possible limitation is that the majority of patients answering the survey were women (80%). Indeed, male patients are also less likely to attend medical appointments. This may have affected the result, although no association was found between sex and hesitancy in those who answered the survey.

In regards to its web-based nature, older people may have been less likely to complete the survey because they may be less familiar with digital technology and the internet. Consequently, the majority of the survey respondents may have consisted of younger patients familiar with IT. However, it is not known whether this group of patients is more or less receptive to vaccination compared with the entire cohort.

Lastly, a matched control group was not recruited from the general population. To overcome this limitation, we compared vaccine hesitancy of our CD patients with a control group of 12,322 Italians from the general population, albeit not crudely matched [19].

Nevertheless, our study has many strengths. First, these are the first published data on acceptance of COVID-19 vaccination among patients with CD, which is one of the most common chronic conditions affecting mankind with a prevalence usually reported to be about 1% in the general population. [34] Second, Lombardy, where the study was carried out, was the first and most badly affected European region, and so our respondents were likely to be fully aware of the severity of COVID-19. 

## 5. Conclusions

Our study showed that most CD patients would accept a COVID-19 vaccine, although one in every four is hesitant or refuses at the present time. 

Since CD patients should be encouraged to receive all common vaccines, including those against COVID-19, the authors believe that identification of the reasons and determinants influencing patients’ attitude to COVID-19 vaccines is of great importance for the scientific community and for public health officials, so that specific vaccination campaigns can be developed and patient–doctor communication optimized.

## Figures and Tables

**Table 1:** Sociodemographic, lifestyle and clinical characteristics of CD respondents.

Characteristic	(*N* = 103)
Age (years), median (range)	48 (18–77)
Female, *n* (%)	81 (78.6%)
Marital status, *n* (%)	
Single/divorced/widowed	77 (74.8%)
Married or cohabiting	26 (25.2%)
Number of family members, *n* (%)	
≤2 Members	41 (32.3%)
>2 Members	86 (67.7%)
Children under 10 years of age, *n* (%)	15 (14.5%)
Educational level, *n* (%)	
Undergraduate	46 (44.7%)
Graduate	57 (55.3%)
Healthcare professionals, *n* (%)	8 (7.8%)
Alcohol intake, *n* (%)	57 (55.3%)
Active lifestyle, *n* (%)	65 (63.1%)
Vegetarian or vegan diet, *n* (%)	5 (4.9%)
Positive attitude to CAM, *n* (%)	9 (8.7%)
Adherence to therapy, *n* (%)	
Yes	96 (93.2%)
No/not at all	7 (6.8%)
Smoking habit	
Smoker/former smoker	10 (9.7%)
Never Willingness to be vaccinated Willing Hesitant	93 (90.3%) 77 (74.8%) 26 (25.2%)

*CAM* complementary and alternative medicines; *CD* celiac disease. Active lifestyle was intended as a self-reported regular physical activity.

**Table 2:** Crude OR and adjusted OR (AdjOR) association analysis of sociodemographic, lifestyle and clinical characteristics, knowledge, attitudes and perceptions about COVID-19 and general vaccination with willingness to be vaccinated against COVID-19 in patients with CD.

Characteristic	Crude OR	95% CI	*p* Value	AdjOR	95% CI	*p* Value
Age in years (continuous variable)	1.00	(0.96–1.04)	0.91			
Gender						
Female	Ref		0.17	Ref		0.13
Male	2.5	(0.68–9.31)		3.58	(0.67–19.03)	
Marital status						
Single/divorced/widowed	Ref		0.77			
Married or cohabiting	0.85	(0.30–2.43)				
Number of family members						
≤2 Members	Ref		0.76			
>2 Members	1.15	(0.47–2.83)				
Children under 10 years of age						
No	Ref		0.39			
Yes	0.60	(0.18–1.95)				
Educational level						
Undergraduate	Ref		0.86			
Graduate	1.08	(0.44–2.64)				
Smoking habit						
No	Ref		0.72			
Yes	0.77	(0.18–3.21)				
Alcohol intake						
No	Ref		0.78			
Yes	0.88	(0.35–2.16)				
Active lifestyle						
No	Ref		0.50			
Yes	1.36	(0.55–3.37)				
Vegetarian or vegan diet						
No	Ref		0.78			
Yes	1.37	(0.14–12.84)				
Positive attitude to CAM						
No	Ref		<0.05	Ref		0.70
Yes	0.23	(0.57–0.95)		0.70	(0.11–4.34)	
Disease duration						
<5 Years	Ref		0.97			
>5 Years	0.97	(0.18–5.14)				
Adherence to GFD						
No/not at all	Ref		0.17	Ref		<0.05
Yes	3.17	(0.59–16.81)		12.71	(1.82–88.58)	
Previous negative experience (personal, family members, relatives) of vaccination						
No	Ref		0.07	Ref		0.76
Yes	0.37	(0.13–1.07)		0.80	(0.18–3.44)	
Attitude regarding vaccination						
Negative	Ref		<0.001	Ref		<0.01
Positive	2.39	(3.47–34.3)		16.48	(3.34–81.31)	
Perceived higher risk of contracting COVID-19 due to CD						
No	Ref		0.71			
Yes	1.23	(0.40–3.75)				
Perceived higher risk of negative effects related to vaccination due to CD						
No	Ref		<0.05	Ref		0.08
Yes	0.27	(0.09–0.78)		0.29	(0.07–1.16)	
Perception of a good knowledge about COVID-19 and its vaccines						
No	Ref		<0.05	Ref		<0.05
Yes	4.05	(1.33–12.37)		6.50	(1.44–29.22)	

*CAM* complementary and alternative medicines; *GFD* gluten free diet; *CD* celiac disease; Active lifestyle was intended as a self-reported regular physical activity.

**Table 3:** Knowledge, attitudes, and perception about COVID-19 in CD patients.

Characteristic, *n* (%)	(N = 103)
Perceived higher risk of contracting COVID-19 due to CD	22 (21.4%)
Perceived more severe COVID-19 due to CD	27 (26.2%)
Perceived higher risk of COVID-19 vaccine adverse events due to CD	19 (18.4%)

*CD* celiac disease.

## Data Availability

The datasets generated during and/or analyzed during the current study are not publicly available but are available from the corresponding author on reasonable request.

## Supplementary Materials

The following are available online at https://www.mdpi.com/article/10.3390/vaccines9050511/s1, Table S1: Questionnaire.
