# Supplementary material for: COVID-19 Vaccine: A Survey of Hesitancy in Patients with Celiac Disease
Source: Vaccines (Basel). 2021 May 16;9(5):511. doi: 10.3390/vaccines9050511 (PMC8156726; doi:10.3390/vaccines9050511)
Supplement: Supplementary file 1 [file vaccines-09-00511-s001.zip › vaccines-1226731-supplementary.pdf]

Table 1. Questionnaire.

A) Sociodemographic information and educational level, including gender, age, nationality, level of education, marital status, parental status and work activities, specifically if healthcare providers.
B) Information regarding the course of celiac disease, in terms of disease subtype (e.g., refractory celiac disease, non-celiac gluten sensibility), illness duration, therapies and adherence to a gluten-free diet.
C) Lifestyle, health-related behaviors and attitudes, including smoking, physical activity, approach to screening services.
D) Knowledge and perceptions regarding vaccination and vaccine-preventable diseases.
E) Vaccination history.
F) Sources of information on vaccines, such as general practitioner, the mass media, pharmacists.
G) Reports by people close to the respondent regarding vaccines and adverse events.
H) Personal opinion regarding the association between celiac disease and COVID-19, in terms of risk of infection and disease severity.
I) Personal opinion regarding the association between celiac disease and COVID-19 vaccines, perceived as increased risk of adverse events.
J) Intention to get the COVID-19 vaccine.
